# Health effects of indebtedness: a systematic review

**DOI:** 10.1186/1471-2458-14-489

**Published:** 2014-05-22

**Authors:** Elina Turunen, Heikki Hiilamo

**Affiliations:** 1 Research Department, The Social Insurance Institution of Finland, PL 450, 00101 Helsinki, Finland; 2 Department of Social Research, Social and Public Policy, University of Helsinki, P.O. Box 16, 00014 Helsinki, Finland

**Keywords:** Indebtedness, Mental health, Depression, Suicidal behaviour, Mortality, Physical health, Health-related behaviour, Debt counselling

## Abstract

**Background:**

In the aftermath of the global financial crisis, millions of households have been left with debts that they are unable to manage. Indebtedness may impair the wellbeing of those affected by it for years to come. This systematic review focuses on the long-term consequences of indebtedness on health.

**Methods:**

The method used in the paper is a systematic review. First, bibliographic databases were searched for peer-reviewed articles. Second, the references and citations of the included articles were searched for additional articles.

**Results:**

The results from our sample of 33 peer-reviewed studies demonstrate serious health effects related to indebtedness. Individuals with unmet loan payments had suicidal ideation and suffered from depression more often than those without such financial problems. Unpaid financial obligations were also related to poorer subjective health and health-related behaviour. Debt counselling and other programmes to mitigate debt-related stress are needed to alleviate the adverse effects of indebtedness on health.

**Conclusions:**

The results demonstrate that indebtedness has serious effects on health.

## Background

In the aftermath of the global financial crisis, millions of households in the Western world have been left with debts that they are unable to manage [1,2]. Indebtedness may impair the wellbeing of those affected by it for years to come. The effect of unpaid household debts has been the subject of much recent research. However, so far no systematic literature review has been available.

On a general level, household debt is not a sign of financial problems. On the contrary, most households take out loans to finance housing purchases and other types of private consumption, in some cases also for private investments and businesses. Loans are granted based on the borrower’s ability to pay back the loans. However, if a household’s financial problems accumulate, for example as a result of unemployment, severe illness, the collapse of property values or rising interest rates, it may no longer be able to manage its debts and the existing financial problems will become more severe.

There is no uniform definition for indebtedness (or over-indebtedness). The condition where a household falls behind in its loan payments and cannot escape the legal consequences of unmet financial obligations is generally referred to as indebtedness. Existing measures of consumer indebtedness are largely based on pragmatic grounds [3]. According to Betti et al. [3], the measures can be classified based on the following general models: (i) the administrative model, (ii) the “objective” (or quantitative) model, and (iii) the subjective model. The administrative model examines all cases where a non-payment of debts has been registered officially or declared before a court. The objective model devises quantitative measures that try to capture the net indebtedness or the debt service burden of households (e.g. debt to assets, debt to income and/or credit scores) and then establishes threshold levels for the ratios that are regarded as abnormally high and that will put consumers in danger of becoming indebted. Under the subjective model, indebted consumers are classified as those who consider themselves to be indebted. In this systematic review, we define those individuals who repeatedly are unable to meet their loan payments as indebted.

Indebtedness can be defined as a lack of possible debt redemption in due time, resulting in a remarkable cutback in a household’s standard of living [4]. Unpaid consumer debts come with various social consequences. First, households may lose access to the lines of credit that would otherwise be available to them if they had no problems with interest rates and loan payments. They may also encounter problems in finding rental apartments or re-employment. Second, households with unpaid loans become subject to various debt-collection actions, including foreclosure. The legal consequences of unmanageable debts vary greatly in different countries. However, we may still assume that the financial distress caused by indebtedness is a common risk factor for morbidity and mortality.

Economic crises can have devastating health effects. Indebtedness concerns the financial security of those affected by it. Higher debt loads may induce stress and the existing debt burden may hinder the borrower from making health-maximizing choices and cause individuals to work harder to maintain their debt service, thereby producing additional stress [5]. A lack of financial resources may lead to unhealthy coping mechanisms, while at the same time individuals may be tempted to cut back on the costs of health care and medicines. Taking on more debt to survive financial problems may exacerbate the problem and again have potential adverse effects on health. The health effects of indebtedness could, in turn, weaken an individual’s ability to get out of debt. Creditors might also have a strong interest in getting paid. Referring to the United States, Jacoby [5] notes that informal debt collection leaves creditors free to demand full payment and to settle for nothing less.

The main aim of this systematic review is to summarise the consequences of indebtedness on mental and physical health. Specifically, we focus on how indebtedness affects mental health, depression, suicidal behaviour, mortality, physical health and health-related behaviours. Different social, cultural and political contexts affect the connections between indebtedness and the studied outcomes. We concentrate on seeking evidence from studies carried out in developed, Western democratic countries.

## Methods

The data were collected in two stages. First, bibliographic databases were searched for peer-reviewed articles on the effects of indebtedness on health between November 2012 and February 2013. Second, the list of references of the included studies was used to locate additional peer-reviewed articles using the specified search terms. The elementary search included the following databases: PsycINFO, PubMed, SocINDEX with Full Text, SpringerLink, Wiley Online Library, EconLit, Worldwide Political Science Abstracts, Sociological Abstracts, Social Services Abstracts and ASSIA: Applied Social Sciences Index and Abstracts. The time of the publication of the studies was not specified in this search.

The following English terms were used in the search: “debt”, “indebtedness”, “over-indebtedness”, “bankruptcy”, “mortality”, “suicide”, “death”, “morbidity”, “mental health”, “depression”, “stress”, “physical health” and “health behaviour”. (A list of the search terms is presented in List 1.) To gain access to a wider pool of research, we also included search terms in German and Swedish. The German terms included “Schuld”, “Überschuldung”, “Schuldnerberatung”, “Schuldenberatung”, “schuldenbelastet”, “Kredit”, “Konkurs” and “Bankrott”. The Swedish terms included “skuld” and “skuldsatt”.

List 1 Search terms for studies focusing on the health consequences of indebtedness

a) debt OR indebtedness OR over-indebtedness OR bankruptcy

b) (debt OR indebtedness OR over-indebtedness OR bankruptcy) AND (mortality OR suicide OR death)

c) (debt OR indebtedness OR over-indebtedness OR bankruptcy) AND morbidity

d) (debt OR indebtedness OR over-indebtedness OR bankruptcy) AND (mental health OR depression OR stress)

e) (debt OR indebtedness OR over-indebtedness OR bankruptcy) AND (physical health OR health behaviour)

f) Schuld OR Überschuldung OR Schuldnerberatung OR Schuldenberatung OR schuldenbelastet OR Kredit OR Konkurs OR Bankrott

g) skuld OR skuldsatt

Electronic libraries searched [November 2012 – February 2013]: PsycINFO, PubMed, SocINDEX with Full Text, SpringerLink, Wiley Online Library, EconLit, Worldwide Political Science Abstracts, Sociological Abstracts, Social Services Abstracts and ASSIA: Applied Social Sciences Index and Abstracts [searches (f) and (g) were done without PsycINFO and EconLit].

1. The initial search with the above mentioned search terms resulted in 1,874 abstracts to be subject to the following inclusion/exclusion criteria.

Studies were included if

a) the focus was on debt and morbidity or mortality (types of outcome measures);

Studies were eliminated if

b) the studies did not deal with developed countries (types of participants);

c) the results were derived from qualitative inquiry (types of studies);

d) the studies were not peer-reviewed.

2. The process resulted in 61 abstracts (1,813 abstracts were eliminated from the study). Complete articles were obtained for the abstracts.

3. The above mentioned inclusion/exclusion criteria were applied to full text articles whereby 42 articles were eliminated and we were left with a total of 19 studies.

4. The list of references in the 19 articles was searched using the specified search terms (List 1) to locate additional peer-reviewed articles. After applying the above-mentioned inclusion/exclusion criteria for the cited articles, 14 additional papers were identified.

5. Thus, the final sample included 33 peer-reviewed publications. The publication years in the sample stretched from 1994 to 2013.

Both authors were involved in selecting the articles and discussing borderline cases. No disagreements emerged. Both authors were involved in analysing the selected articles.

The studies in the final sample applied different definitions of indebtedness and studied a wide range of morbidity and mortality outcomes. The studies were located in different countries and some of them were nationally representative and some not, and some were concentrated on the household and some on the individual level. The studies had different data sources as well as sample sizes and age groups. The sample included 30 studies which were based on survey data and three register-based studies. Due to the heterogeneity of the sample, the quality of the studies and the study designs could not be assessed systematically beyond the above mentioned inclusion/exclusion criteria.

## Results

The results of the review are presented in Additional file 1: Table S1. With respect to the geographical composition of our sample, most of the research on the health consequences of indebtedness was conducted in the US (48% of the studies). Approximately one-third of the studies were carried out in Great Britain (the second largest area). The rest of the studies in the sample were from Germany, Finland, Austria and Australia. The majority of the studies were carried out in the 2000s (55%), eleven began or were carried out in the 1990s, and two studies were from the 1980s. One study focused on developments in the 19th and 20th centuries.

Fourteen of the studies were based on nationally representative samples (see Additional file 1: Table S1). The size of the study population varied from 106 to 27,131 individuals. Some of the articles were based on longitudinal studies [6-17]. All of the articles applied quantitative methods, most using different regression analysis techniques.

The target populations and definitions of indebtedness varied across the studies. Three articles concerned older individuals [18,19,7], whereas one article dealt with young adults [20], two of them dealt with students [21,22] and one study dealt with married couples [6]. One article pertained to households with at least some outstanding debt [23], and one of them pertained to people on the verge of bankruptcy [24]. One study dealt with individuals seeking assistance with outstanding debt and who had joined a debt management programme in the US [15]. Three studies concerned indebted individuals who sought out debt counselling agencies in Germany [4,25,26].

The measures for indebtedness varied across the sample. A study by Lenton and Mosley [11], for instance, comprised both objective and quantitative measures of assets, income and expenditures as well as subjective self-assessments of measures pertaining to wellbeing and responses to questions about feelings and attitudes. Collateralized debt was dealt with in five studies [7,27,12-14] and non-collateralized debt in six studies [28,6,30,22,18]. However, most of the studies covered both collateralized and non-collateralized debt. Indebtedness was studied on the individual level in twenty-one studies and on the household level in twelve studies.

The studies covered debts from different sources. Medical debt was discussed in one study [29], mortgage in five studies [7,27,12-14] and borrowing money from a friend or relative or increasing credit card debt in one study [28]. Credit card debt was specifically focused on in two studies [30,22]; in one of the studies, Drentea and Lavrakas [30] also observed the financial strain resulting from any debt. Consumer debt and out-of-pocket medical expenses were discussed in one study [18].

The analysis most often included age, race/ethnicity, gender, education, income, marital status, employment status, number of children/family size/household size, health insurance status or Medicaid eligibility and housing status (including homeownership/housing tenure/household tenure) as confounding factors.

We will review the results from our sample based on six themes: the effects on mental health, depression, suicidal behaviour, mortality, physical health and health-related behaviour.

### Effects on mental health

The onset of mortgage indebtedness appears to be associated with deteriorating mental health [14]. Persons who failed to pay their mortgage or whose house was repossessed reported a particularly high prevalence of mental and physical health impairments [27]. They experienced poorer health relative to homeowners with no housing strain for every measure examined, including the number of days in the past month during which time mental health was impaired (other measures include self-rated health, psychological distress, the number of days in the past month during which time physical health was impaired and physical symptoms). McLaughlin et al. [12] also found foreclosure to be associated with an increased rate of symptoms pertaining to major depression and generalized anxiety disorder.

Lenton and Mosley [11] found that both psychological and physical health was affected by debt. Two intermediating variables influence the linkages: a high-interest debt repayment structure and worry exacerbate debt problems and influence health-seeking behaviour. Financial concern was also a significant linear predictor of mental and physical health in a study conducted by Jessop, Herberts and Solomon [21]. Increased financial concern was consistently associated with worse health. It appeared to mediate all of the negative impacts of the amount of debt on health per se.

Those experiencing difficulties in repaying their debts had some sort of minor mental disorder more often than those without such difficulties [10]. Being seriously behind on any debt repayment and particular sources of debt were key correlates of all common types of mental disorders, both as a whole and when analysed by separate diagnostic criteria categories for mental disorders.

Generally, financial stress was found to be higher in families with more children, with more income units and dependents and with an older head of the household as well as among families from ethnic minorities [23]. This was especially the case when families were reliant on government pensions and benefits. Financial stress was found to be lower in families with higher disposable incomes and housing values.

For female heads of households, longer-term unsustainable housing commitments had psychological costs [13]. These effects were in addition to and larger in magnitude than those associated with financial hardship more generally. For male heads of households, arrears and housing payment problems had significant psychological costs over and above those related to the associated negative financial events [13]. The experience of the onset of mortgage indebtedness for men was associated with increased rates of consultation with general practitioners [14].

Financial strain had less effect on mental health if the individual had strong self-efficacy beliefs, a belief in his or her own competence, a belief in his or her ability to cope, and greater access to some sort of collective purpose [24]. However, having more access to social contacts was related to better mental health only if the perceived financial strain was low. In a study by Selenko and Batinic [24], employment status had little effect on the relationship between financial strain and mental health.

Individuals use various strategies to cope with the financial burden caused by the high cost of prescription medication. Martin et al. [28] found that those using the cost-coping strategy of borrowing money had worse psychosocial health and greater disability. Those with mounting credit card debt reported worse physical functioning and self-rated health and a greater sense of helplessness. The cost-coping strategy of medication underuse was associated with worse psychological health, greater disability and depressive symptoms.

The direction of causation for indebtedness and mental health is likely to run in both directions [31]. In a study by Meltzer et al. [31], there was no multiplicative effect of debt and addictive behaviours, so it looks as though addictive behaviours have an effect on common mental disorders apart from debt. Patients with debt were more likely to receive a psychiatric diagnosis [32].

### Effects on depression

Self-reported problems of indebtedness and financial stress were strongly associated with depression [8], and indebtedness was also associated with depression-related symptoms such as anxiety and anger [19]. Mortgage delinquency and defaulting on a housing loan (borrower had given a lien on the property as collateral for the loan) were associated with a significant elevation in the incidence of depressive symptoms, food insecurity and cost-related medication non-adherence [7]. The relationship between debt and mental health was explained by financial strain [19].

Income correlated strongly with depression outcomes in a study done by Zimmerman and Katon [20]. When other variables (education, employment status, the ratio of debt to assets, job type, insurance status, home ownership) were controlled for, income lost much of its statistical significance. Current employment status and the ratio of debt to assets were more robust predictors of depression. However, instrumental variable estimates suggested that financial strain may not lead to depression. Another study found that the effect of income on mental illness appears to be mediated largely by debt [33].

Debtor status, i.e. those having any debt (credit cards, store credit, a mortgage or home equity loan, a car loan or any other loan), was more consistently associated with mental health than any other single traditional indicator of socioeconomic status [19]. Also, Lee and Brown [18] found that financial distress factors, such as higher consumer debt and lower retirement wealth, were significant predictors of depressive symptoms for both older women and men. For example, having higher medical expenses, having lower net worth, being widowed, having less education, having poorer perceived health and fair/poor or good health increased the level of depressive symptoms.

Key drivers for the onset of depression included a general perception of financial difficulties, job loss and worsening physical health [8]. In addition, Gathergood [9] noted that much of the cross-sectional variation in problem debt (that is, the inability to repay debts) and psychological health was attributable to omitted variables and selection. Exogenous factors (such as negative local house price movements) made the consequences of problem debt more severe. They impacted the extent of deterioration in psychological health when homeowners had late housing payments or a heavy burden of consumer credit. Respondents’ reactions to problem debt also had a noticeable social dimension. The prevailing rate of indebtedness in a given geographical area impacted individual psychological stress. Relatively less stress was associated with a higher rate of indebtedness.

Researchers have also found that among mothers with infants, there was a close relationship between financial hardship and depressed mood [34]. Worries about debt appeared to be the strongest predictor of a depressed mood.

### Effects on suicidal behaviour

Indebted individuals were found to have greater suicidal intent [32], and debt was also found to be a factor independently associated with suicidal ideation [35,10]. Meltzer et al. [35] found that gender, age, marital status, employment status, alcohol, gambling, relationship break-ups or problems, being bullied, a financial crisis, and having something valuable lost or stolen increased the odds of suicidal ideation. Hopelessness was one main mediator between debt and suicidal ideation, although it was also independently associated with suicidal behaviour.

The causal link may also work the other way around. Kidger et al. [17] found that individuals admitted to a trauma centre following an attempted suicide were more than twice as likely to become bankrupt within two years compared to those who were admitted following an accident. The relationship between attempted suicide and pre-injury bankruptcy was weaker, but still visible, particularly when bankruptcy was restricted to Chapter 7 (in the US bankruptcy laws) liquidation cases.

Weyerer and Wiedenmann [16] studied the effects of four economic variables (economic growth, average real income, unemployment and frequency of bankruptcy) on suicide rates in Germany between 1881 and 1989. The strongest correlations held true for the rate of unemployment and for the frequency of bankruptcy in times of obvious social disintegration and diminished social safeguards provided by the state. However, the differences among all correlation values were not significant. Economic factors did not influence male suicide rates more strongly than female suicide rates.

### Effects on mortality

Brzoska and Rasum [36] studied the effects of individual indebtedness on death rates in 439 administrative districts in Germany. They found that indebtedness and unemployment correlated slightly with mortality. Indebtedness, taken together with the unemployment rate, income, the ratio of new companies and population density, explained 59% of the variance in district-level mortality, while indebtedness alone explained 6% of it.

### Effects on physical health

The state of health of indebted persons was markedly subnormal [26]. The evidence presented in a study by Münster et al. [26] suggests that two mechanisms interact with one another: indebtedness leads to illness and illness leads to indebtedness. However, the study could not provide causal explanations for the risk factors and incidence of diseases.

In a study by Drentea and Lavrakas [30], the ratio of credit card debt to total family income was significantly associated with worse physical health and worse self-reported health. Some of the relationships between the debt to income ratio and increased impairment in both physical and self-reported health were explained by financial strain. Credit card debt had a stronger effect than income on health in the physical health analysis. The health-related behaviours and risks explained part of the relationship between debt, financial strain and health. In the case of self-reported health, the strength of the relationship between the debt-to-income ratio and health decreased and became non-significant, but the effect of financial strain on health remained significant. In the case of physical impairment, the strength of the relationship between the debt-to-income ratio and physical impairment decreased. Havlik, Vukasin and Ariyan [37] found that there was a significantly higher occurrence of bankruptcy or unemployment and divorce or marital separation in the five years prior to the clinical presentation of 56 melanoma patients relative to a control group. Twenty per cent of melanoma patients had sustained a major financial crisis involving bankruptcy or unemployment prior to clinical presentation. This may relate to increased health care-seeking behaviour or reflect an increased susceptibility of the patient following major psychosocial stress.

Kim, Garman and Sorhaindo [15] found that credit counselling (assistance given to consumers with financial troubles) indirectly affected financial wellbeing and health. Financial wellbeing was influenced by financial behaviours and financial stressor events. Credit counselling reduced the financial stressor events of clients who stayed in the programme for 18 months.

### Effects on health-related behaviour

The health-seeking behaviour of people was influenced by the high-interest debt repayment structure and worry, both of which exacerbated debt problems [11]. Poorer health behaviours were related to a higher probability of having non-collateralized debt, though these estimates cannot be interpreted as causal [6]. Grafova [6] showed that between 1999 and 2003 in the US, non-collateralized debt was not associated with non-specific psychological distress, but this did not entirely rule out the possibility that non-collateralized debt could cause poorer health behaviours through anxiety and frustration.

Herman, Rissi and Walsh [29] found that the insured status of a person and medical debt were both independent predictors of delayed access to care, but only medical debt predicted whether an individual would delay or forgo medications. McLaughlin et al. [12] noted that the high rates of unemployment, financial strain and lack of health insurance coverage among those experiencing foreclosure might limit access to mental health services, and predatory lending practices targeted at low-income areas could exacerbate health problems.

Drentea and Lavrakas [30] found that health-related behaviours and risks explained some of the relationships between debt, financial strain and health. Indebtedness was associated with an increased prevalence of being overweight and obesity [4] as well as back pain [25].

In a study by Nelson, Lust, Story and Ehlinger [22], credit card debt of at least 1,000 USD was found to be a more robust indicator of unhealthy weight-related behaviours compared to either high perceived stress or poor stress management, which were not statistically significant. Weight-related behaviours included physical activity, sedentary behaviour (such as watching TV), dietary patterns, unhealthy weight-control behaviours and body satisfaction. Credit card debt of at least 1,000 USD and poor stress management significantly predicted risk behaviours of engaging in physical fights, binge drinking and using tobacco, marijuana and/or other drugs.

## Discussion

The results based on this systematic literature review reveal the serious health effects of indebtedness. Individuals with unmet loan payments had suicidal ideation and suffered from depression more often than those without such problems. Unpaid financial obligations were also related to poorer subjective health and health-related behaviours. The connections between indebtedness and poor health are not clear-cut. Instead, they are influenced by factors such as source of debt, collateral status, repayment structure and interest rates. Employment status, the value of assets and personality traits act as mediating factors.

Among wealthier countries, the causal link between indebtedness and health seems to run through a mental process where indebted individuals suffer from emotions of shame and failure. These emotions, referred to as financial strain (or worry or concern), may weaken mental health and lead to coping behaviour that is detrimental to health. However, a lack of financial resources may also result in medication underuse [28].

The source of debt had little effect on the prevalence of common mental disorders [31], though some types of debt were reported more often than others among people with a mental disorder [33]. Regarding the relationship between the sources of debt and suicidal ideation, the largest odds ratios were found for debts relating to several different categories (shopping, housing and utilities) and for those relating to just the one category of shopping debt [35].

According to Meltzer et al. [31], borrowing from moneylenders might be psychologically more difficult to deal with than borrowing from friends or family, which could be because of the high interest rates attached to the debt in the first case. Those who borrowed from moneylenders had the highest rate of common mental disorders, while Lenton and Mosley [11] have demonstrated that being faced with a low-interest repayment structure, i.e. having access to “cheaper” methods to finance immediate debts, significantly increased the probability of reporting good health. Being faced with high interest credit had a negative, though insignificant direct impact, on reports of good health. There was also an increased impact of high-interest debt on worry, and it was much stronger and more significant for lower income groups. Thus, the multiplier effect that worry adds to the impact of debt on health was greater for those least able to control or manage that debt.

Lenton and Mosley [11] argue that worry interacts with debt structure, thereby constraining the decision space available to low-income people to pursue health-seeking behaviour. Worthington [23] found that higher repayment amounts on loans to buy or build owner-occupied property were associated with lower financial stress. The number of credit cards that a person owned was also associated with lower financial stress. It is likely that better access to credit cards increases financial flexibility and therefore has a role in diminishing financial stress in all but the most extreme circumstances.

Less research was found on the connections between indebtedness and specific physical impairments. Ochsmann et al. [25] noted that psychological variables were probably related to the onset of pain and to the occurrence of acute, sub-chronic and chronic pain. Financial strain was probably linked to perceived poor health and depression and might influence the prevalence of back pain via these mechanisms, too. Indebted persons threatened by personal bankruptcy were more likely to complain about back pain than the general population. Havlik, Vukasin and Ariyan [37] suggested that the fact that a fifth of all melanoma patients had experienced bankruptcy or unemployment before the clinical presentation might be related to increased health care-seeking behaviour or reflect an increased susceptibility of the patient following major psychosocial stress. Drentea and Lavrakas [30] found that part of the relationship between indebtedness and health was explained by financial strain. The results indicate that the effects of indebtedness are first seen in mental health, and therefore they are over-represented in the research.

Given the heterogeneity of the sample, it was not possible to systematically evaluate the quality of the reviewed studies. Therefore no assessment of risk of bias in individual studies was undertaken. It was not always clear if the studies had adequately controlled for measurement errors or selection bias [9], i.e. factors predicting indebtedness before it occurred. It is also possible that poor health causes debt problems rather than that debt problems cause poor health [17,26,31,32]. The studies in our sample did not apply a universal definition of indebtedness. Such a definition seems unattainable due to the culturally and legally bound implications of indebtedness and due to variations in the consumer credit market.

We were not able to pay attention to the timing of the reviewed studies with regard to business cycles, which may play a role in the connection between indebtedness and health. Large-scale economic shock affecting a large portion of the population may carry less of a social burden for indebted individuals than experiencing such problems during an economic upswing [2]. It is possible that the literature search was biased. It was not possible to use the thesauruses available in the search engines, which would have provided a tailor-made way to focus on the themes discussed here. The extent to which synonyms are covered in the search is important, and it is possible that not all of synonyms for indebtedness were used.

More research that controls for selection bias and applies a uniform definition of indebtedness is clearly needed to unravel the connections between indebtedness and health. The body of evidence already available suggests, though, that debt counselling and other programmes to mitigate debt-related stress serve an important public health function.

## Conclusions

The results from our sample of 33 peer-reviewed studies demonstrate the serious health effects of indebtedness. Individuals with unmet loan payments had suicidal thoughts and suffered from depression more often than those without such problems. Unpaid financial obligations were also related to poorer subjective health assessments and health-related behaviour. Thus, indebtedness might have serious and long-lasting impacts on people’s lives.

## Competing interests

The authors declare that they have no competing interests.

## Authors’ contributions

ET has designed the literature review, acquired and analysed the literature, and drafted and edited the manuscript. HH has participated in the design of the study, participated in selecting and analysing the studies, and drafted and revised the manuscript critically. Both authors read and approved the final manuscript.

## Pre-publication history

The pre-publication history for this paper can be accessed here:

http://www.biomedcentral.com/1471-2458/14/489/prepub

## Supplementary Material

Additional file 1: Table S1 Included studies. Click here for file
