# Supplementary material for: Health effects of indebtedness: a systematic review
Source: BMC Public Health. 2014 May 22;14:489. doi: 10.1186/1471-2458-14-489 (PMC4060868; doi:10.1186/1471-2458-14-489)
Supplement: Additional file 1: Table S1 — Included studies. [file 1471-2458-14-489-S1.docx]

**Additional file 1: Table S1.** Included studies

| Study Type | Year(s) | Study Population | Nationally representative | Method | Measure | Outcome | Adjustment for | Study Conclusions |
| --- | --- | --- | --- | --- | --- | --- | --- | --- |
| Alley, Lloyd, Pagán, Pollack, Shardell & Cannuscio, 2011 [7] | 2006, 2008 | A nationally representative sample of 2,474 individuals in the USA, older than 50 years of age. | yes | Logistic regression | Mortgage delinquency. | Depressive symptoms, self-rated health, medication adherence and lack of  money to buy necessary  food or receive food stamps. | Demographic and socioeconomic indicators, smoking status, chronic conditions, self-reported physical activity. | Mortgage delinquency associated with significant elevations in the incidence of depressive symptoms, food insecurity and cost-related medication non-adherence. |
| Bridges & Disney, 2010 [8] | 1999, 2001, 2005 | 4,659 low-income households with children (1999–) and 8,062 households with children  (2001–) in Britain. | no | Univariate and bivariate probit model, fixed-effect logistic model | Indebtedness. | Psychological well-being. | Demographic and socioeconomic indicators, health, children with chronic health problems. | Strong association between being in a depressed psychological state and self-reported problems of indebtedness and financial stress. Key drivers for the onset of depression are a general perception of financial difficulties, job loss and worsening ill-health. |
| Brzoska & Rasum, 2008 [36] | 2002–2003 | 439 administrative districts in Germany. | yes | Multiple linear regression | Individual indebtedness. | Death rate. | Population density, number of hospital beds, the ratio of residents to new companies,  the proportion of candidates  for the matriculation examination, unemployment rate, personal income. | Indebtedness and unemployment slightly correlated. The unemployment rate, indebtedness, income, the ratio of new companies and population density explain 59% and indebtedness 6% of the variance in district-level mortality. |
| Cannuscio et al., 2012 [27] | 2008 | 798 individuals in Arizona, California, Florida and Nevada, USA. | no | Logistic regression, negative binomial regression | Housing strain (homeowners with no housing strain, with moderate housing strain, those in default or foreclosure, and renters). | Self-rated health,  psychological distress, the number of days within the last 30 days in which physical or mental health was impaired, physical symptoms. | Demographic and socioeconomic indicators. | Persons with home in default or foreclosure reported a particularly high prevalence of mental and physical health impairments. Homeowners in default or foreclosure experienced poorer health relative to homeowners with no housing strain. |
| Drentea & Lavrakas, 2000 [30] | 1997 | 970 adults in Ohio, USA. | no | OLS hierarchical regression | Credit card debt, debt stress index. | Self-reported health, physical functioning, body mass index, smoking, drinking. | Demographic and socioeconomic indicators. | The debt/income ratio was significantly associated with worse physical health and self-reported health, while financial strain partially explained the latter association. Credit card debt has a stronger effect than income on physical health. The health-related behaviours and risks explained part of the relationship between debt, financial strain and health. |
| Drentea & Reynolds, 2012 [19] | 2002–2003 (unclear) | 1,463 individuals in Miami-Dade County, Florida, USA. Older individuals over-represented. | no | Logistic regression | Indebtedness. | Symptoms of depression, anxiety, anger. | Demographic and socioeconomic indicators, physical disability status,  early health problems,  financial strain. | Indebtedness associated with more symptoms of depression, anxiety and anger. The association between debt and mental health explained by financial strain. |
| Gathergood, 2012 [9] | 1991–2008 (annual) | A nationally representative sample of approximately 5,500 households in Britain (1991). Number of observations 66,664 (1991–2008) and 54,731 (1995–2008). | yes | Panel regression, second-state regression | Indebtedness. | Psychological state (GHQ12 Score, anxiety-related illness). | Demographic and socioeconomic indicators,  local-level mortgage and consumer credit delinquency, psychological health of the spouse, house price changes, reference group effects (repossession rate &  bankruptcy rate). | Much of the cross-sectional variation in problem debt and psychological health is attributable to omitted variables and selection. Exogenous factors (local house price movements and local level of indebtedness) have an impact on respondents’ psychological stress. |
| Grafova, 2007 [6] | 1999, 2001, 2003 | A nationally representative sample of US individuals. 2,499 (1999), 2,442 (2001)  and 2,651 (2003) households. Married couples (retirees excluded). | yes | Probit, multinomial probit and fixed-effects OLS regression | Smoking, psychological distress, Body Mass Index (BMI). | Non-collateralized debt  (NCD). | Demographic and socioeconomic indicators,  total out-of-pocket medical expenditures. | Poorer health behaviours are related to a higher probability of having NCD. NCD not associated with non-specific psychological distress, but there is a possibility that NCD could cause poorer health behaviours through anxiety and frustration. |
| Hatcher, 1994 [32] | 1989 | 147 individuals in Britain. | no | Cross tabulation | Financial indicators, threats of court action, help sought for money, to what extent worry about money contributed to self-harm. | Self-poisoning. | Age, employment state,  past and present psychiatric treatment, follow-up arrangements and diagnosis, history of deliberate self-harm. | There was a high prevalence of debt affecting more than one-third of patients. Those in debt had a greater rate of suicidal intent. |
| Havlik, Vukasin & Ariyan, 1992 [37] | 1983–1987 | 56 new melanoma patients  and a control group between 20 and 88 years of age in Connecticut, USA. | no | Cross tabulation | Divorce or separation, unemployment or bankruptcy, death of spouse or family  member. | Clinical presentation of melanoma. | None. | There was a significantly higher occurrence of divorce or marital separation and bankruptcy or unemployment in the 5 years prior to the clinical presentation of 56 melanoma patients relative to a control group. This may be related to increased health care-seeking behaviour or reflect an increased susceptibility of the patient following major psychosocial stress. |
| Herman, Rissi & Walsh, 2011 [29] | 2008 | 2,368 adults in Arizona,  USA. | no | Logistic regression | Medical debt, insurance status. | Access to medications and medical care (delaying or obtaining). | Demographic and socioeconomic indicators,  health status, Medicaid eligibility, usual source of  health care, perceived  value of care or medication. | Insured status and medical debt are both independent predictors of delayed access to care, but only medical debt predicts whether an individual will delay or forgo medications. |
| Hintikka, Kontula, Saarinen, Tanskanen, Koskela & Viinamäki, 1998 [10] | 1993, 1994, 1995 | A nationally representative sample of 4,868 individuals from the general Finnish population. | yes | Univariate and multivariate logistic regression | Indebtedness. | Suicidal behaviour. | Demographic and socioeconomic indicators, increase in family problems, alcohol abuse. | Those experiencing difficulties in repaying their debts more often than others had a probable minor mental disorder. Difficulties in repaying debts independently associated with suicidal ideation. |
| Jenkins, Bhugra, Bebbington, Brugha, Farrell, Coid, Fryers, Weich, Singleton & Meltzer, 2008 [33] | 2000 | A nationally representative survey of 8,580 individuals in Britain. | yes | Logistic regression | Low income. | Mental disorder (psychosis, neurosis, alcohol and drug abuse). | Demographic and socioeconomic indicators. | Low income and debt are both associated with mental illness, but the effect of income appears to be mediated largely by debt. |
| Jessop, Herberts & Solomon, 2005 [21] | unclear | 89 British and 98 Finnish students. | no | Hierarchical and standard multiple regressions | Amount of debt,  financial concern. | Mental and physical health. | Demographic and socioeconomic indicators, smoking and drinking  behaviour, missing lectures  due to work, perception of control. | Financial concern a significant linear predictor of mental and physical health, increased financial concern being associated with worse health. It appeared to mediate any negative impact of the amount of debt per se on health. |
| Kidger, Gunnell, Jarvik, Overstreet & Hollingworth, 2011 [17] | 1991–2004 | 27,131 injured individuals in Washington, USA. | no | Univariable and multivariable logistic regression | Attempted suicide. | Personal bankruptcy in the 2 years following and prior to emergency department admission. | Demographic and socioeconomic indicators, Medicaid eligibility, prior bankruptcy, details concerning injury. | Individuals in a trauma centre following an attempted suicide were more than twice as likely to become bankrupt within 2 years compared to those in a centre following an accident. The association between attempted suicide and pre-injury bankruptcy was weaker, but visible. |
| Kim, Garman & Sorhaindo,  2003 [15] | 2000, 2002 | A random sample of 175 credit counselling clients in the USA. | no | Path analysis, regression analysis | Financial behaviours, financial stressor  events, credit counselling. | Perceived financial  well-being, health. | Age, income. | Credit counselling effective in decreasing financial stressor events of clients who stayed in the programme for 18 months, but the effects on financial behaviours not significant; an indirect effect of credit counselling on financial well-being and health. |
| Lee & Brown, 2007 [18] | 2000 | A subsample of 8,845 individuals of a nationally representative sample of the population in the USA, aged 65 or older. | yes | Ordinary Least Squares regression | Consumer debt, out-of-pocket medical expenses, net worth, income. | Depressive symptoms. | Demographic and socioeconomic indicators,  self-reported health, chronic health condition. | Financial distress factors significant predictors of depressive symptoms for both older women and men. |
| Lenton & Mosley, 2008 [11] | 2003, 2004, 2005 | 5,717 heads of households  in Britain. | no | Random-effects ordered probit model, random effects regression model, simultaneous-equation generalized probit model | Indebtedness, debt repayment structure and worrying, health seeking behaviour, lagged health. | Health, indebtedness, total value of debt, worrying  about debt. | Demographic and socioeconomic indicators, disability status, social  capital, membership  in a credit union or savings group. | Psychological and physical health affected by debt. Two intermediating variables, high-interest debt repayment structure and worry, exacerbate debt problems and influence health-seeking behaviour. |
| Martin, Shreffler, Schoster & Callahan, 2012 [28] | 2006 | 729 individuals in North Carolina, USA. | no | Multivariate  logistic and linear regression | Cutting back on necessities, medication restrictions, borrowing money from a friend or relative, increasing  credit card debt. | Health assessment questionnaire (HAQ),  physical and mental health functioning, self-rated health, depressive symptoms, helplessness. | Demographic and socioeconomic indicators,  body mass index, comorbid condition count. | Participants borrowing money had worse psychosocial health and greater disability. Those with increasing credit card debt reported worse physical functioning, self-rated health and greater helplessness. Medication underuse associated with worse psychosocial health, greater disability and depressive symptoms. |
| McLaughlin et al., 2012 [12] | 2008–2009, 2010 | A probability sample of 1,547 predominantly African-American adults in Michigan, USA. | no | Logistic regression, Poisson regression | Home foreclosure. | Symptoms of major depression and generalized anxiety disorder (GAD). | Demographic and socioeconomic indicators, history of psychiatric disorder, exposure to other financial stressors. | Foreclosure associated with an increased rate of symptoms of major depression and GAD. |
| Meltzer, Bebbington, Brugha, Farrell & Jenkins, 2013 [31] | 2007 | A random probability sample of 7,461 respondents selected for a national survey in Britain. | yes | Univariate and multivariate logistic regression | Debt. | Common mental disorders (CMD). | Demographic and socioeconomic indicators, alcohol dependence, drug use and dependence, problem gambling. | Debt and particular sources of debt are key correlates of all CMD, both as a whole and analysed separately. The direction of causation likely to run in both directions. No multiplicative effect of debt and addictive behaviours on CMD. |
| Meltzer, Bebbington, Brugha, Jenkins, McManus & Dennis, 2011 [35] | 2007 | A random probability sample of 7,461 respondents selected for a national survey in Britain. | yes | Univariate and multivariable logistic regression | Personal debt. | Suicidal ideation. | Demographic and socioeconomic indicators, lifestyle behaviours, adverse experiences, hopelessness. | Debt and particular sources of debt are key correlates of suicidal behaviour. Hopelessness one main mediator between debt and suicidal ideation, also independently associated. |
| Münster, Rüger, Ochsmann, Alsmann & Letzel, 2007 [26] | 2006–2007 | 666 over-indebted persons  in Germany. | no | Cross tabulation | Indebtedness. | Current diseases. | Gender. | The state of health of over-indebted persons is markedly subnormal. The study cannot provide causal explanations for risk factors and the incidence of diseases. |
| Münster, Rüger, Ochsmann, Letzel & Toschke, 2009 [4] | 2003; 2006–2007 | A survey of 8,139 subjects representative of the German population and a cross-sectional study of 941 over-indebted individuals in Germany. | no | Multiple logistic regression | Over-indebtedness. | Overweight and obesity. | Age, sex, education,  income, body-mass-index, depression, smoking  behaviour. | Over-indebtedness associated with an increased prevalence of being overweight and obesity. |
| Nelson, Lust, Story & Ehlinger, 2008 [22] | 2004 | A random sample of 3,206 college students in  Minnesota, USA. | no | Poisson regression | Credit card debt, stress level, ability to manage stress. | Body mass index, physical activity and sedentary behaviour, dietary intake, unhealthy weight control behaviours and body satisfaction, other risk behaviours. | Gender, race, year in school, hours per week working  for pay. | Credit card debt of at least 1,000 USD is a more robust indicator of unhealthy weight-related behaviours compared with a high perceived stress or poor stress management (not significant). The same amount of debt and poor stress management significantly predicted risk behaviours (engagement in physical fights, binge drinking and use of tobacco and other drugs). |
| Nettleton & Burrows, 1998 [14] | 1991–1992,  1994–1995 | A subsample of 3,700 and 3,500 (second-period) individuals in a nationally representative random sample of the population in Britain. | yes | Multiple regression, logistic regression | Mortgage indebtedness. | Mental health, use of general practitioners. | Household income and employment status, physical health problems, residential mobility, new child, ended relationship. | The onset of mortgage indebtedness associated with the deterioration of mental health. The experience for men also associated with increased rates of consultation with general practitioners. |
| Ochsmann, Rüger, Letzel,  Drexler & Münster, 2009 [25] | 2003; 2006–2007 | A survey that included 8,318 individuals representative of the German population and a cross-sectional study that included 949 over-indebted individuals in Germany. | no | Multivariate binary logistic regression | Over-indebtedness. | Back pain. | Demographic and socioeconomic indicators, physical activity, smoking behaviour, body mass index, mental illnesses/depression. | Over-indebted individuals might suffer from back pain more often than individuals from the general population. Over-indebtedness might be an independent moderator variable. |
| Reading & Reynolds, 2001 [34] | 1997–1998 | 271 families in Britain. | no | Ordinary least-squares regression, multiple regression | Debt, worries about  debt and money. | Maternal depression. | Demographic and socioeconomic indicators, variables on social support  and networks, child health. | A close relationship between financial hardship and depressed mood in mothers of infants. Worries about debt appeared to be the strongest predictor, but it was also associated with the depression score and with receiving benefits (worries removed from the equation). |
| Selenko & Batinic, 2011 [24] | 2009 | 106 people on the verge of bankruptcy in Austria. | no | Hierarchical linear regression | Perceived financial  strain. | Mental health. | Demographic and socioeconomic indicators,  latent benefits (social contacts, time structure, collective purpose, status, activity), self-efficacy beliefs. | Financial strain had less effect on mental health if an individual had strong self-efficacy beliefs and more access to a collective purpose. More access to social contacts related to better mental health only if the perceived financial strain was low. Employment status had little effect on the relationship. |
| Taylor, Pevalin & Todd, 2007 [13] | 1991–2003, annually | A subsample of a nationally representative random sample of the population in Britain: 5,651 male and 2,534 female heads of households. | yes | Multivariate, fixed-effects regression models | Housing payment problems and arrears  (rent or mortgage). | Mental health. | Demographic and various socioeconomic indicators. | For male heads of households, arrears and housing payment problems have significant psychological costs, over and above those relating to the associated negative financial events. For female heads of households, longer-term unsustainable housing commitments have psychological costs. |
| Weyerer & Wiedenmann, 1995 [16] | 1881–1939,  1949–1989 | Germany | yes | Pearson correlations | Growth of the domestic product, real income, unemployment, frequency of bankruptcy. | Rates of suicide. | Social disintegration. | The strongest correlations held true for the rate of unemployment and for the frequency of bankruptcy on the rates of suicide in times of obvious social disintegration and diminished state social safeguards, though the differences among all correlation values are not significant. |
| Worthington, 2006 [23] | 1998–1999 | A nationally representative sample of 3,268 households with at least some outstanding debt in Australia. | yes | Binary logit regression | Demographic and socioeconomic  indicators, debt repayment of various types and credit card usage. | Financial stress  (relatively mild forms). |  | Financial stress is higher in families with more children, income units and dependents, older heads of households and those from ethnic minorities, and lower in families with higher disposable incomes and housing values. Debt had a weak but significant influence on financial stress, which is offset elsewhere. |
| Zimmerman & Katon, 2005 [20] | 1992 | Based on a nationally representative  sample of 8,489 young adults in the USA. | yes | Fixed-effects logit, instrumental variables probit and negative binomial regressions | Income. | Depression. | Demographic and socioeconomic indicators, physical health problems (past/present), self-esteem. | Income correlates strongly with depression outcomes. When other variables are controlled, it loses much of its relationship. Other variables, such as current employment status and the ratio of debt to assets, are more robust predictors of depression. However, instrumental variable estimates suggest that financial strain may not lead to depression. |
